# Osteopathic Orthopaedic Residency Selection Criteria: Program Directors’ Survey and Analysis

**DOI:** 10.51894/001c.11598

**Published:** 2020-01-30

**Authors:** Michael McDonald, Saad Khan, Clarence Cabatu, Fremont Scott

**Affiliations:** 1 Orthopedic Surgery Henry Ford Macomb Hospital; 2 Orthopedic Traumatologist Advocate Medical Group; 3 Orthopedic Surgery Henry Ford Macomb; 4 Orthopedic Surgery Program Director Henry Ford Macomb

**Keywords:** osteopathic, orthopaedic, orthopedic, selection, criteria, survey, residency, program director

## Abstract

**CONTEXT:**

Orthopaedic Surgery has become one of the most competitive specialties. Each year the number of applicants is far greater than the number of available Orthopaedic residency training spots [1,2,3]. With medical schools expanding their class sizes and new medical schools opening out of proportion to the number of residency spots, the competition is becoming even more fierce [12]. There are several published articles on resident selection in allopathic orthopaedic programs [5-7]. However, there are currently no such published studies on osteopathic orthopaedic programs to our knowledge. With the AOA and ACGME merger, this topic is critical to both allopathic and osteopathic applicants alike. The goal of our study was to evaluate the resident selection criteria for osteopathic orthopaedic residency programs.

**METHODS:**

A twenty-five-question survey was sent to all of the osteopathic orthopaedic programs in December of 2017. The most important selection factors were then calculated as a mean of all the responses and were ranked accordingly.

**RESULTS:**

The survey was completed by 29 out of 41 program directors (71%). The most important factors in resident selection were performance during the student’s rotation at the program, formality/politeness and performance in the interview, and medical school board exam scores.

**CONCLUSIONS:**

This study is the most comprehensive study to date on the osteopathic orthopaedic resident selection process. The results from this study will help future applicants, both MD and DO, to focus on the factors in resident selection. The results may also help programs evaluate their own selection process and make improvements.

## INTRODUCTION

Orthopaedic Surgery is now one of the most competitive residency program specialties. The United States Medical Licensing Examination (USMLE) and Comprehensive Medical Licensing Examination (COMLEX) scores of matched orthopaedic applicants are amongst the highest in all clinical specialties.^1,2^ The orthopaedic surgery training placement model is currently divided into two pathways: one for Allopathic and one for Osteopathic medical school graduates. In 2017, there were 165 allopathic training programs, offering 727 orthopaedic residency spots.^3^ During this same year, there were 1,013 applicants for these allopathic training spots, averaging 1.4 applicants per available spot.^3^

For the 2017 match, there were 41 osteopathic orthopaedic residency programs, offering 121 spots.^4^ Although there are no readily available data regarding the number of applicants to these programs in 2017, there were 187 applicants for 104 available training spots in 2014, roughly 1.8 applicants per spot.^2^

The resident selection process is one of the most discussed topics amongst medical students. However, there is little accurate data available on what orthopaedic programs are prioritizing in applicants, leaving most medical students unaware of what may be considered more important by selection committees.^5,6^

There have been several published studies that have investigated the resident selection criteria for allopathic programs.^5–7^ Bernstein et al.^5^ reported that an orthopaedic clerkship at their institution and USMLE Step I to be of utmost importance to allopathic program directors. Orr et al.^7^ further investigated the selection criteria for a series of US Army orthopaedic residency programs and reported orthopaedic clerkship performance at their institution and USMLE Step I and II exams were often the most important factors.

In addition, processes for resident selection have been studied, with proposed guidelines for optimal resident selection criteria published, most often placing emphasis on USMLE scores, honors societies, and class rank.^8–10^ Such studies have been helpful for those attempting to understand the process of resident selection as applicants and program administrators. Although these results are available for allopathic programs, there are no such studies concerning osteopathic program resident selection.

The osteopathic orthopaedic community is smaller, and there remains a lack of clear and accurate information on what is important in the residency selection process. It has been widely recognized that many osteopathic orthopaedic program directors value earlier clerkship rotation performance at their program as the most important factor. Many orthopaedic programs will not interview applicants unless they have done a medical school rotation at their institution. The authors were unable to locate any previous literature concerning whether osteopathic orthopaedic programs value the same application aspects as their allopathic counterparts.

However, there have been some changes with the recent expansion of osteopathic orthopaedic residencies.^2,4^ The American Osteopathic Association (AOA) and the Accreditation Council for Graduate Medical Education (ACGME) are in the process of merging to create one accreditation system for both allopathic and osteopathic orthopaedic surgery residency programs. With this impending merger, it has been concluded that program directors may be putting more emphasis on licensing exam scores and research activities, factors that may have been previously been considered less important than applicants’ rotation performance.^11^

**Purpose:** The purpose of this study was to evaluate current osteopathic orthopaedic program directors’ resident selection criteria, specifically which factors they perceived to be of most importance. The authors also attempted to evaluate the interview processes at responding osteopathic orthopaedic programs. Finally, the authors assessed the program directors’ perceptions concerning future allopathic applicants to their programs.

## METHODS

The authors emailed a 25-question survey questionnaire to 41 osteopathic orthopaedic program directors across the country in December of 2017. Google Forms surveying software was utilized to design an electronic questionnaire based on reported common themes in orthopaedic resident selection. Google Forms is software that allows you to create and email custom questionnaires. The survey consisted of 17 selection variables (Table 1), most of which had been used in previous studies to evaluate applicant selection criteria. The variables utilized in our questionnaire were selected based on previous studies looking at orthopaedic resident selection specifically, as well as variables that our home institution finds valuable.^5–8^ Program directors were asked to rank the variables from 1 to 10, with 1 being the “least important” and 10 being the “most important.”

The survey questionnaire also included eight multiple choice and Yes/No items to further evaluate the selection criteria, understand respondents’ interview processes and assess their attitudes on the AOA-ACGME merger (Table 2). Participants were assured that the survey was anonymous; no personal or identifying information was asked or shared.

The survey was sent to all 41 osteopathic program directors across the country. The mean was calculated for each selection variable as rated by program directors.

**Table 1.** Orthopaedic Resident Selection Criteria: Survey Questionnaire

On a scale of 1-10 please rate the importance you give to each of the following factors in selecting a candidate for residency. Please give an importance rating to each qualification.
Importance Rating Scale *No Importance 1 2 3 4 5 6 7 8 9 10 Utmost Importance*
COMLEX Level I/ USMLE Step I COMLEX Level II/ USMLE Step II Candidate Has rotated at your Program Candidate has Published Research Candidate has Dedicated Research experience Letter of recommendation from a known Orthopaedic Surgeon Telephone call made on behalf of candidate Dean’s letter Personal Appearance of the Candidate on the interview day Formality/Politeness of the Candidate on the interview day Performance on Ethical questions during interview Personal Statement Candidate has/had a relative affiliated with the institution Inability to get into an Orthopaedic program on initial attempt Reputation of medical school Receipt of Thank you letters Sigma Sigma phi

**Table 2.** Program Director Survey Multiple Choice Questions

1. The most important aspects of a letter of recommendation are that: The letter is written by an orthopaedic surgeon The letter is written by a well-known orthopaedic surgeon The letter is overwhelmingly positive The letter is written by someone that I personally know 2. The most important aspect of a personal statement is: To gain insight into the applicant’s decision to pursue orthopaedics To gain insight into the applicant’s writing and communication abilities To learn more about the candidate’s personal interests and background I do not feel that the personal statement is very important or valuable in evaluating a candidate 3. The interview process at our institution can best be categorized as: Straightforward process with the goal of getting to know the applicant Emphasis on problem solving and/or manual skills Emphasis on ethical issues Emphasis on psychological testing 4. Your interview process includes manual skills testing. Yes No 5. Your interview process includes presentations of clinical scenarios for assessment of the applicants. Yes No 6. Once selected for an interview, all candidates are considered as equal for the final decision made based solely on the candidate’s performance during the interview. Yes No 7. Residents' opinion is heavily weighted in resident selection. Yes No 8. Will you consider MD candidates once achieving ACGME accreditation? Yes Yes, but only if they rotated at our program Yes, but will favor DO candidates No, MD candidates should only apply to allopathic programs

## RESULTS

The survey questionnaire was returned by 29 (71%) out of 41 program directors. Each respondent answered all questions and ranked each of the selection factors.

**Table 3.** Results of 17-Item Survey Questionnaire (N = 29)

**Mean of Program Directors' Responses**	**Rank**	**Residency Selection Criteria**
9.36	1	Rotation at Program Director's Institution
7.94	2	Formality/Politeness in interview
7.91	3	Performance on ethical questions in interview
7.89	4	COMLEX I/USMLE Step I
7.68	5	COMLEX II/USMLE Step II
7.47	6	Personal appearance on interview day
6.78	7	Failed first attempt at Orthopaedic Match
6.63	8	Letter from a Known Orthopaedic Surgeon
6.26	9	Published research experience
5.89	10	Dedicated Research experience (No publication)
5.68	11	Telephone call made on behalf of candidate
5.57	12	Personal Statement
4.01	13	Dean's Letter
3.89	14	Reputation of medical school
3.68	15	Candidate has a relative affiliated with the program
3.31	16	Thank You letter from candidate
2.84	17	Sigma Sigma Phi membership

**Table 4.** Results of Program Director Multiple Choice Question Survey

The most important aspects of a letter of recommendation are that: The letter is written by an orthopaedic surgeon (20.6%) The letter is written by a well-known orthopaedic surgeon (10.3%) The letter is overwhelmingly positive (17.4%) The letter is written by someone that I personally know (51.7%) The most important aspect of a personal statement is: To gain insight into the applicant’s decision to pursue orthopaedics (6.9%) To gain insight into the applicant’s writing and communication abilities (17.2%) To learn more about the candidate’s personal interests and background (37.9%) I do not feel that the personal statement is very important or valuable in evaluating a candidate (37.9%) The interview process at our institution can best be categorized as: Straightforward process with the goal of getting to know the applicant (86.2%) Emphasis on problem solving and/or manual skills (6.9%) Emphasis on ethical issues (6.9%) Emphasis on psychological testing (0%) Your interview process includes manual skills testing. Yes (20.7%) No (79.3%) Your interview process includes presentations of clinical scenarios for assessment of the applicants. Yes (72.4%) No (27.6%) Once selected for an interview, all candidates are considered as equal for the final decision made based solely on the candidate’s performance during the interview. Yes (37.9%) No (62.1%) Currently Training Residents' opinion is heavily weighted in resident selection. Yes (89.7%) No (10.3%) Will you consider MD candidates once achieving ACGME accreditation? Yes (31%) Yes, but only if they rotated at our program (24.1%) Yes, but will favor DO candidates (17.3%) No, MD candidates should only apply to allopathic programs (27.6%)

The most important resident selection factor was overwhelmingly a clerkship rotation at the institution with an average response of 9.36, with 27 (93.1%) of responding program directors ranking this as their most important selection factor (Table 3). Candidates’ formality/politeness as well as their performance on ethical questions were ranked second highest. Following closely were scores in licensing exams, both COMLEX/USMLE I and II respectively. The least important factor ranked in orthopaedic residency selection was involvement in the Sigma Sigma Phi honor’s society.

In the multiple choice section of the survey, over half (51.7%) of the respondents reported that a letter of recommendation from someone they knew was the most important selection factor (Table 4). More than one-third of respondents (37.9%) reported that they didn’t feel the applicants’ personal statements were important or valuable in evaluating them, although the same number of respondents (37.9%) used personal statements to learn more about their background and interests (Table 4).

The majority (86.2%) indicated that the residency interview was a straight forward process at their institution with the goal of getting to know the applicant (Table 4). Most respondents (79.3%) reported that manual skills testing was not a routine part of interviews at their institution (Table 4). However, clinical scenarios (i.e., brief descriptions of a **clinical** situation or event) were also used for assessment of applicants at most programs (72.4%) (Table 4).

Most (62.1%) respondents indicated that all candidates were not considered as equally qualified applicants based solely on their interview performance. The vast majority (89.7%) of respondents also used their currently training residents’ opinion heavily during applicant selection (Table 4).

In addition, 31% of respondents indicated that they would consider allopathic candidates without any stipulations, although 24.1% indicated that they would only consider those applicants who had earlier rotated through their program (Table 4). A total of six (17.3%) respondents reported that they would consider allopathic candidates but would prefer osteopathic applicants, and over quarter (27.6%) of the program director respondents indicated that they will not consider allopathic candidates (Table 4).

## DISCUSSION

Resident selection processes continue to be integrally important for both the orthopaedic residency programs and applicants. Whether an orthopaedic residency program applicant will turn into an increasingly expert orthopod remains a highly subjective topic that has been studied with mixed results.^6–10,13^ Several previous studies with allopathic programs have reported that a clerkship rotation at their residency program setting is considered the most important factor in resident selection.^5–7^ Our study was conducted to identify the most important variables that affect an applicant’s residency selection through the perspective of a national convenience sample of program directors.

In our study, clerkship performance rankings were quite closely followed by USMLE Step 1 scores and performance during medical school. The largest difference among programs was between the top two variables (i.e., clerkship rotation and candidate interview formality/politeness), demonstrating that the osteopathic program directors’ respondents may have relied more heavily on students’ clerkship rotations to evaluate applicants (Table 3).

Evaluating applications continues to be a time consuming and multifactorial process. Our study results indicate that a letter of recommendation was generally considered to be of high importance, with 51.7% of respondents indicating that a letter from someone the program directors personally knew was more heavily weighted than letters coming from a well-known orthopaedic surgeon (10.3%) (Table 4). This is consistent with results from Bernstein et al. who found that letters from orthopedic surgeons that were personally known by the program director was important.^5^ It should be noted that respondents considered indicated a phone call made on behalf of an applicant ranked more highly than their personal statements or Dean’s letters (Table 3).

This finding suggests that it may be of paramount importance for osteopathic medical students to find a mentor early, one who can not only write a letter of recommendation but be willing to make a phone call on the applicant’s behalf.

One of the more surprising aspects of our study was the perceived importance of published research to osteopathic program directors. Published and nonpublished research experience were ranked 9 and 10 respectively out of the 17 items (Table 3). When comparing data available from an earlier 2002 allopathic program resident selection study, a Dean’s letter, personal statement, and medical school reputation were ranked more highly than this “published research” item.^5^ This may be reflective of ACGME residency review committees now requiring increased resident and faculty research participation and encouraging published research.^13^

The osteopathic program directors in this sample placed a relatively high emphasis on the interview process and applicants’ personalities, as evident by high ranking of formality/politeness and performance on ethical questions during the interview. The majority of participants (86.2%) indicated that the interview process at their institution was a straight forward process with emphasis on getting to better know the applicant (Table 4).

One of the most underrecognized aspects of the resident selection process in the literature may be the use of feedback from the programs’ current residents. Since current residents work closely with students during their clerkship, they are often in a good position to evaluate an applicant’s work ethic, knowledge, and character than interviewing faculty (Table 4). With the AOA-ACGME merger to be completed in 2020, there is still some uncertainty about the increasingly competitive resident selection process in current osteopathic programs after 2020.^7,8^ To investigate this controversial topic, we also asked respondents about their perceptions of future allopathic applicants to their historically osteopathic programs.

A total of nine (31%) of respondents indicated that they would consider allopathic applicants without stipulation, with 24.1% reporting that they would only consider allopathic applicants who had earlier rotated through their program. At the same time, five (17.3%) respondents concluded that they would still prefer osteopathic applicants. Surprisingly, a relatively large percentage (n = 8, 27.6%) of sample program directors indicated they would not consider allopathic applicants (Table 4).

Study Limitations

The biggest limitation to our study is the number of responding osteopathic orthopaedic program directors. Another limitation of our study is that we only asked program directors to participate. Furthermore, those in leadership positions at orthopaedic residency programs can change frequently and affect the residency program selection criteria from year to year.

## CONCLUSIONS

Applying for a competitive specialty such as orthopedic surgery can be a daunting process for medical students. Not only can the process be stressful and time consuming, but it also imposes significant economic costs to applicants.^14,15^ Providing accurate information to applicants on how to increase their potential for matching into orthopaedic surgery will continue to be an important topic.

Ideally, the results from this study can help current students as well as future applicants, both allopathic and osteopathic, to focus on the more important selection criteria at an early stage. Based on these results, it is important for most applicants to establish relationships with faculty and residents at their desired orthopaedic residency programs.

Attention should be focused on performing well during away rotations, interpersonal skills, and board scores. It may also prove important for allopathic applicants to inquire whether certain osteopathically-oriented residency programs will consider allopathic student applicants. Ideally, these results may also be used by program officials to improve their selection process criteria and to make further changes as competition increases.

### Prior Poster presentations of these overall results:

1. McDonald M, Khan S, Scott F, Cabatu C. Osteopathic Orthopedic Residency Selection Criteria: Program Directors’ Survey and Analysis. American Osteopathic Academy of Orthopedics Fall Meeting 2018.
2. McDonald M, Khan S, Scott F, Cabatu C. Osteopathic Orthopedic Residency Selection Criteria: Program Directors’ Survey and Analysis. Statewide Campus System Scholarly Activity Poster Day 2018

### Conflict of Interest

The authors declare no conflict of interest.
